# Random Telegraph Noise in 3D NAND Flash Memories

**DOI:** 10.3390/mi12060703

**Published:** 2021-06-16

**Authors:** Alessandro S. Spinelli, Gerardo Malavena, Andrea L. Lacaita, Christian Monzio Compagnoni

**Affiliations:** Dipartimento di Elettronica, Informazione e Bioingegneria, Politecnico di Milano, 20133 Milan, Italy; gerardo.malavena@polimi.it (G.M.); andrea.lacaita@polimi.it (A.L.L.); christian.monzio@polimi.it (C.M.C.)

**Keywords:** 3D NAND Flash memories, random telegraph noise, Flash memory reliability

## Abstract

In this paper, we review the phenomenology of random telegraph noise (RTN) in 3D NAND Flash arrays. The main features of such arrays resulting from their mainstream integration scheme are first discussed, pointing out the relevant role played by the polycrystalline nature of the string silicon channels on current transport. Starting from that, experimental data for RTN in 3D arrays are presented and explained via theoretical and simulation models. The attention is drawn, in particular, to the changes in the RTN dependences on the array working conditions that resulted from the transition from planar to 3D architectures. Such changes are explained by considering the impact of highly-defective grain boundaries on percolative current transport in cell channels in combination with the localized nature of the RTN traps.

## 1. Introduction

Random telegraph noise (RTN) in MOS transistors has been an important topic of interest in the solid-state device community since the 80s, when results of low-frequency noise characterization [1] showed a transition from a typical 1/f behavior at high temperatures to a series of discrete switching events as temperature was lowered. Similar observations were soon made when moving from large- to small-area devices [2], and interpreted in terms of capture/emission of electrons by single interface traps. On the theoretical side, this result highlighted the importance of the number fluctuation contribution to the flicker noise, but prompted the emergence of a new limitation to MOS device operation as well [3].

Moving from early investigations and models [4,5,6,7,8,9], the RTN picture grew more complex, as novel time and amplitude observations [10,11,12,13,14] hinted at a non-negligible role played by non-uniform electron conduction in submicron devices [15,16]. This idea gained traction when the phenomenon began to be investigated in Flash memories [17,18,19,20,21,22,23], demonstrating current fluctuations up to 60% [22] and threshold voltage ($V_{T}$) shifts reaching 700 mV [18] in 90-nm technology node devices. The physical picture now accepted that accounts for such results is based on the fact that, in scaled devices, dopants must be viewed as individual ions rather than a continuous distribution, resulting in randomly-placed charges in the depletion region. Such random point charges [24,25,26,27] give rise to sharp peaks in the band energy profile of the channel of an MOS transistor, resulting in local modulation of the current flow and filamentary conduction. If a “strategic” trap happens to be placed right above a current path, electron trapping will effectively shut off such a path, resulting in a large drain current and $V_{T}$ fluctuation [28,29,30,31]. On the other hand, if the trap is placed over a region in which little current flows, its trapping/detrapping will barely affect the overall current. Such an idea has been successfully applied to explain the statistical distribution of the RTN fluctuations in NOR and NAND arrays, measured in terms of their amplitude [19,32,33] and time constants [34,35,36], providing a useful tool for extracting information about the impact of device parameters on RTN. A recent review of the issue can be found in [37].

The above-mentioned framework has served nicely the Flash community until the first decade of the 21st century, when several limitations to the scaling of the planar NAND technology prompted the emergence of 3D arrays [38]. In such devices, the RTN picture just outlined fell short of adequately describing the experimental data, in view of the peculiar characteristics of the polycrystalline material used as conduction channel.

In the following, we will review in detail the physics of RTN in 3D NAND Flash memories, discussing the main experimental data and physical models developed to quantitatively account for them. We begin our discussion with a brief summary of the main 3D array architecture and cell structure, followed by a description of electron transport in 3D NAND channels. This will allow us to develop a consistent picture of RTN in 3D NAND devices, whose main features will be highlighted. After this part, we will focus our attention on the main experimental data presented in the literature, taking advantage of the model results to provide interpretation for them.

## 2. Array and Cell Structure

Among the several architectural solutions for 3D storage [39,40,41,42,43,44,45,46], the one employing vertical-channel strings crossed by a set of planar wordlines has become the most effective one [47,48,49,50], and is the focus of this section. Here we will briefly describe the main features of such an array, namely its organization and cell structure, referring to previous works for further details [51].

**Figure 1:**
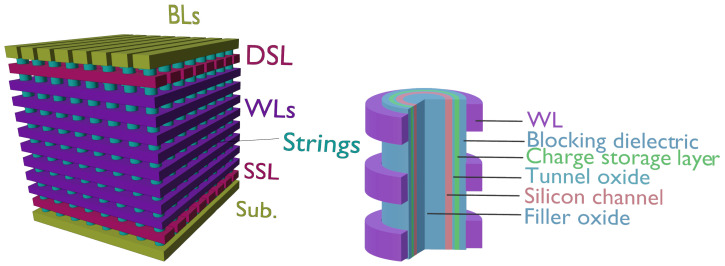
(**Left**) Conceptual view of a vertical-channel 3D NAND array with its main elements (SSL = source select line, WLs = wordlines, DSL = drain select line, BLs = bitlines). (**Right**) pictorial view of an array string highlighting the structure of the elementary memory cells.

A pictorial view of the array is shown in Figure 1 (left): note that the cell strings run vertically from the substrate to the bitlines. As in planar arrays, select elements are needed near the source and drain ends of the string, integrated in rows running orthogonally to the bitlines. The rest of the cells are contacted by planar wordlines that span over an entire block of the array. One of the advantages of this structure is that the large increase in density allowed by the exploitation of the third dimension makes it possible to relieve some of the pressure on channel length scaling and its many drawbacks from the viewpoint of process complexity and reliability, well known in planar devices [52,53]: cell length in 3D NAND is around 25–30 nm [54], with the additional advantage of becoming less dependent on the availability of advanced lithography tools. A second advantage of this solution lies in its manufacturing process: memory cells are not patterned individually, but they are formed all at once as cylindrical holes are cut through the stacked wordlines, creating the strings. This procedure entails that the elementary cell becomes a gate-all-around, vertical-channel transistor, with the advantage of a better electrostatic control from the gate. A schematic view of such a device is shown in Figure 1 (right): starting from the outside we meet a contacted wordline, a blocking dielectric and a charge-storage layer, that can either be a floating gate [47,55,56,57,58,59], similar to planar NAND devices, or a charge-trap layer [60,61,62,63,64], followed by the tunnel oxide. Beyond the oxide, we can notice a thin silicon region and an inner oxide filling the central region of the cylinder, labeled filler oxide for simplicity. This structure, where the conductive channel is a hollow cylinder, is referred to as a “Macaroni” MOSFET, and is the result of clever device engineering in 3D NAND: in fact, after the vertical high-aspect ratio holes have been etched in the structure of Figure 1 (left), and the blocking, storage and tunnel layers deposited, the remaining part of the cylinder must be filled with silicon. The result is a polycrystalline channel whose central region is plagued by a large defectivity, impairing the device performance. To avoid such a drawback, a very thin polysilicon layer is deposited on the gate dielectric, while the remaining central region of the cylinder is filled with a dielectric [40], gaining two distinct advantages: first, thinning of the silicon body results in reduced short-channel effects and better electrostatic control from the gate; second, defect removal further contributes to better subthreshold slope and array performance.

## 3. Polysilicon Conduction

**Figure 2:**
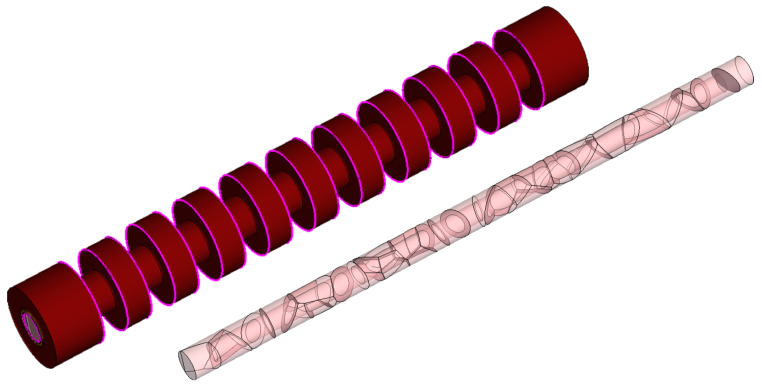
Pictorial view of a ten-cell memory string (**left**) and of the inner polysilicon regions separated into polycrystalline grains (**right**). The example is the result of a TCAD simulation of the cell structure where polysilicon grains are obtained via Voronoi tessellation of the silicon region.

The overview on the memory cell design given in the previous section already suggested that the polycrystalline character of the conduction channel is a key parameter from the viewpoint of device performance. A polycrystalline material, in fact, is formed by single-crystal regions labeled grains, with different crystallographic orientations. Such regions are separated by highly-defective interfaces, or grain boundaries (GBs) [65]. A pictorial view of a NAND string of ten cells with its inner polysilicon region and grains is shown in Figure 2: note the random structure of grains and GBs, that are the key elements affecting device current and variability.

One of the key properties of polysilicon is its trap density, whose value has been estimated by several works, based on either direct optical or electrical experimental measurements [66,67,68,69,70,71,72,73,74] or via numerical device simulations [75,76,77,78,79]. Many of such results point to a double-exponential energy distribution of donor-like and acceptor-like states of the form (for acceptor-like states in the upper half of the energy gap):

(1) $$N_{GB}\left(E\right)=N_{T}e^{-\left(E-E_{C}\right)/E_{T}}+N_{D}e^{-\left(E-E_{C}\right)/E_{D}},$$

where the reported range for the acceptor-like states parameters is listed in Table 1. Note that the first exponential distribution is characterized by a large peak density $N_{T}$ and a small characteristic energy $E_{T}$, and is usually referred to as tail states distribution, as a consequence of its location near the edge of the gap. The second has a lower peak density $N_{D}$ but a higher energy $E_{D}$, and is usually labeled deep states distribution. Note also that trap densities are given as volumetric densities: this was useful in early simulation works, where a uniform trap density in the semiconductor body was assumed for simplicity. From a physical viewpoint, however, traps are expected to be mainly located at GBs, and an areal density σ is then needed. A conversion between volumetric and areal densities is readily achieved assuming for simplicity a spherical grain size with radius $r_{G}$, and placing all volume traps on the sphere surface. This leads to

(2) $$4\pi r_{G}^{2}\sigma =\frac{4}{3}\pi r_{G}^{3}N_{GB}\Rightarrow \sigma =\frac{r_{G}}{3}N_{GB},$$

or a very similar conversion factor as in [79].

Electron transport in polysilicon has been studied since the 70s, as this material found applications in resistors, interconnections, and silicon-gate MOSFETs. From the viewpoint of current conduction, we can identify two modeling approaches, that differ in the way GBs are treated: one approach is to extend the drift-diffusion model usually adopted in monocrystalline silicon, describing GBs as trapping centers with a reduced mobility [80,81,82]; the other is based on a thermionic emission model at the GBs [83,84,85,86,87]. Although the latter seems to be gaining traction in recent literature, a definitive conclusion has not been reached, yet, and a recent study of the different dependences implied by such models can be found in [88,89].

**Figure 3:**
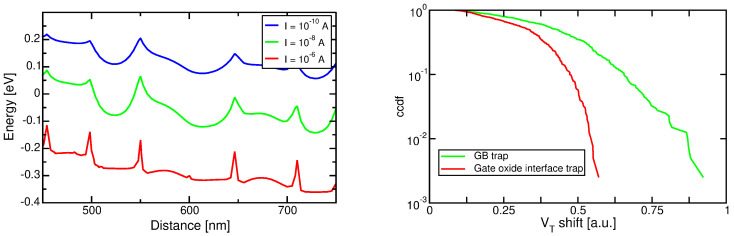
(**Left**) Conduction band profile for a 3D NAND string at different current levels. (**right**) RTN complementary cumulative distribution functions (ccdfs) for traps placed at the GBs or at the gate/oxide interface for a template device.

The above-mentioned numerical models of conduction have been used to investigate the effect of GBs on variability in nanowires [90,91,92,93,94,95] and 3D NAND devices [96,97]. A recent study based on a drift-diffusion transport within the grains and thermionic emission at the GBs [98,99,100,101] has demonstrated a good capability to reproduce several features of experimental data, including its temperature dependence. Figure 3 (left) shows a typical conduction-band profile along the channel of a 3D NAND string, for increasing values of the control-gate bias, as resulting from such model. Note that the profile is not smooth, featuring peaks in correspondence of the highly-defective GBs. As gate bias is increased, the band bending lowers the conduction-band profile, increasing the localized trap occupation and sharpening the peaks, which become the true bottlenecks of conduction [100]. This result makes clear that GBs are an additional source of non-uniformity in the current conduction, which means that they might be expected to play a main role in RTN. This is even more apparent if we consider that GB trap densities (see Table 1) are much larger than typical doping concentrations used in 3D NAND strings. A similar approach was also followed by [102].

The above-mentioned model has been applied to investigate the impact of GB traps on RTN [99,101] within a Monte Carlo approach: random configuration of GBs are first generated in the silicon region after a Voronoi tessellation [92], and traps are placed at the interfaces following the previously-discussed energy distribution. Drain current is computed up to a specified threshold, defined at a constant current level, after which an additional RTN trap is filled with an electron and the resulting $V_{T}$ shift computed. Results for a template device are also reported in Figure 3, for the case of a single trap placed at one random position in a GB, and for a trap placed at a random position at the silicon/gate oxide interface. It is clear that GB traps are much more effective in modulating the electron conduction and result in larger $V_{T}$ fluctuations.

In spite of these encouraging results, several important features of this model still have to be assessed, such as the actual grain size [103,104,105], the mobility degradation and conduction process at the grain boundaries [106,107], and the impact of all these quantities, including architectural parameters and cell design, on RTN.

## 4. Experimental Data

The previous section was meant to provide a framework for the interpretation of the most relevant experimental data presented in the literature, that are discussed in the following. It must be noted, however, that RTN, as well as other reliability concerns in 3D NAND memories, remains a highly-confidential matter and very few data are published. We begin our analysis of RTN with single-trap data, moving then to statistical distributions and impact on device performance.

### 4.1. Single-Trap Data

Investigation of the microscopic properties of RTN single traps in 3D NAND devices can be found in [108,109], where a statistical analysis of the noise power spectral density was also carried out. In those papers it was reported that the string current fluctuations due to single-trap RTN depend on the sensing current: as the current is increased, its fluctuations also increase when measured in absolute terms, but decrease in terms of relative change. Such a dependence was also found in [110] for the above-threshold region, and ascribed to the effect of traps at the silicon-oxide interfaces. These dependences reflect similar behaviors observed both experimentally and numerically in planar or cylindrical devices [111,112,113], where the increased screening exerted by the mobile carriers as the gate bias is raised, mitigating the effect of the RTN trap, was invoked as an explanation. Several works reported investigations of the capture and emission time constants and their dependence on gate bias and temperature, including the activation energies [114,115,116,117]. Their results do not point to any particular difference in the microscopic nature of such traps with respect to those active in planar devices (apart from a faster capture/emission dynamics suggested in [114]): this of course is not surprising and supports an interpretation of the RTN phenomenon based on the spatial distribution of such traps rather than on some peculiar characteristics.

### 4.2. Array Statistical Data

From the viewpoint of the memory performance, the statistical distribution of the RTN-induced $\Delta V_{T}$ is the main parameter. This kind of fluctuations in poly-Si channels were first shown (to our knowledge) in [118], on a nanowire structure (no filler oxide), showing an exponential distribution for $\Delta V_{T}$, which is a typical result of a percolation process. The same exponential dependence was reported on vertical NAND devices in [119,120,121,122,123], suggesting that the RTN distribution in arrays follows an $e^{-\Delta V_{T}/\lambda }$ law, and can be effectively characterized by the slope λ of the exponential distribution.

A comparison between 3D and planar cell RTN is reported in [124], where a larger RTN distribution was reported for the former, while an opposite result was claimed in [121]. It is obviously difficult if not impossible to critically assess those results and search for the reason of this discrepancy. However, from a general standpoint, the slope λ is related to both the trap density (affecting the percolation centers) and the electrostatic impact of a single trapped electron, that have an opposite trend when moving from planar to 3D devices: 3D cells are expected to have a higher trap density thanks to the presence of GBs, but feature also a larger cell (i.e., a larger capacitance and a lower electrostatic impact of a single electron). So, the different results might just be a consequence of different cell designs.

**Figure 4:**
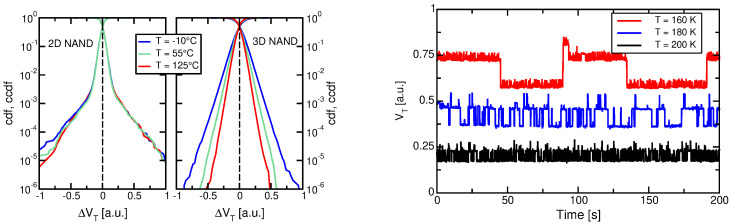
(**Left**) RTN cumulative density function (cdf) and its complementary (ccdf) for 2D and 3D NAND arrays at different temperatures [125,126], © 2017, IEEE. (**Right**) $V_{T}$ fluctuations due to single RTN traps at different temperatures.

The impact of GB traps on RTN can also be noted in the comparison reported in [125,126] and carried out as a function of temperature in the range from −10 to 125 °C, that is shown in Figure 4 (left). First, please note that the shape of the two distributions is different: in 2D cells we notice clear exponential tails due to RTN departing from a central distribution, related to measurement noise in cells not affected by RTN; in 3D arrays, instead, we notice a single exponential distribution, suggesting that the large majority of cells in the 3D array are affected by RTN. A second point to stress is that the slope of the exponential distribution is reduced with respect to planar technologies [121,125,127]. Given the previous point, such an improvement seems mainly a consequence of the larger cell size of 3D arrays, although a role could also be played by the different conduction mechanism and percolation in planar and 3D devices (see for example [113] for a discussion on the RTN dependences in 3D devices). Finally, different temperature dependences are also apparent: while planar device RTN is temperature-independent [128], 3D NAND exhibit a decrease in λ at higher temperatures, as also reported in [115,116].

**Figure 5:**
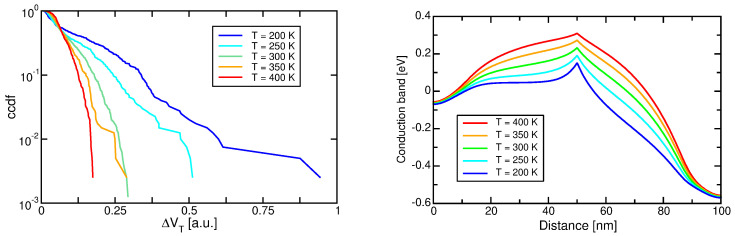
(**Left**) Simulation results for the RTN ccdf in a template 3D NAND device for different temperatures. (**Right**) Conduction band profile at different temperatures for a template device with a single GB located at the center of the channel.

Such a different temperature dependence is important from a reliability standpoint and deserves further investigation. To this aim, the right side of Figure 4 shows the behavior of a single RTN trap as a function of time, for different temperatures. Besides a decrease in the absolute value of $V_{T}$ for higher temperatures, reflecting an increase in the current, it is obvious that the fluctuation amplitude is decreasing as well. This behavior has been observed on a number of traps [126] and is the responsible for the improved RTN distribution. At first glance, the temperature dependence could be simply related to the thermal energy of the electrons and their better or worse capability to overcome the energy barriers, but this would not explain the difference between planar and 3D dependences. So, we must assume that temperature affects the percolation itself. To check this, we conducted simulations with the model presented in the previous section [98,99,100,101], for a template 3D NAND device at different temperatures. Results are presented in Figure 5 (left). Note that the decrease of the RTN slope at higher temperature is accounted for by the model, allowing to exploit its results to provide some more insight: to this aim, we have simulated a template device with a single GB orthogonal to the current flow and placed at the middle of the gate. Results for the conduction band at threshold at different temperatures are reported in Figure 5 (right), and feature significant differences: indeed, the conduction band peak, located at the GB and due to the localized trapped charge, becomes sharper at low temperatures, meaning that there is an increased trapped charge at low temperatures, resulting in more percolation centers. A reason for this lies in our definition of the threshold condition, that is a constant-current (10 nA) criterion. When temperature is lowered, thermal emission is reduced, and the string current lowers. To reach the same 10 nA value, gate bias must be increased, lowering the conduction band and leading to additional trap filling. Note also that this phenomenon does not take place in planar devices, where the percolation centers are the ionized dopants, whose density obviously does not change with the gate bias.

**Figure 6:**
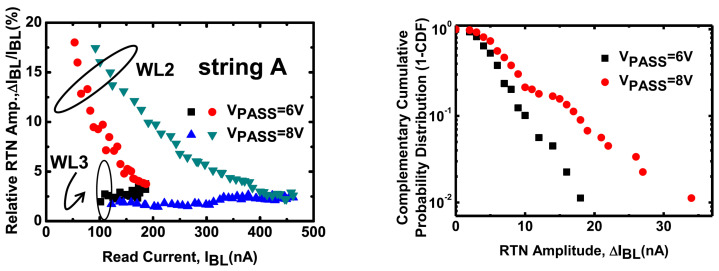
RTN ccdf as a function of the read current (**left**) and of the pass bias, at a read current of 100 nA (**right**). From [122], © 2016, IEEE.

Additional dependences exhibited by random telegraph noise in 3D NAND were reported by [108,122] with reference to read current and pass voltage. Figure 6 (left) shows RTN data for cells on different wordlines as a function of the read current and pass voltage. While some cells do not exhibit significant RTN, the one labelled as WL2 features a decreasing relative fluctuation of the current as the read current is increased, in agreement with data previously discussed. However, data also show a dependence on the pass voltage, whose increase leads to a higher RTN. Similar data are reported in Figure 6 (right), where the RTN distribution is shown. Data show that the tail slope of the bitline current increases as the pass voltage is increased. This result was related to the previous one by the authors of [122], as increasing the pass voltage means a reduction of the read threshold voltage and an increase in the RTN fluctuations. However, further analysis are needed to clarify the link between the string operating conditions and the measured RTN.

**Figure 7:**
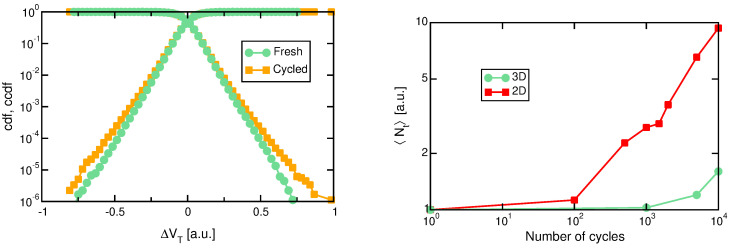
(**Left**) RTN cdf for a 3D NAND before and after cycling to 10k cycles [126], © 2018, IEEE. (**Right**) Average number of RTN traps as a function of cycling [126], © 2018, IEEE.

Finally, the effect of cycling on RTN in 3D arrays has been investigated in [110,119,126] and data from [126] are reported in Figure 7 (left). Note that the RTN $\Delta V_{T}$ data for a fresh and a cycled array show only a minimal increase in the height and slope of the distribution, which is again different from the noticeable increase in the RTN distribution reported in planar devices (see [128,129]). Such a difference can also be appreciated in Figure 7 (right), depicting the average number of traps $\langle N_{t}\rangle$ extracted from fitting the RTN distributions with a simplified model [32]: note that the departing of $\langle N_{t}\rangle$ from the initial value takes place at much higher cycling doses in the 3D NAND case than in the planar array. While this suggests an increased hardening of 3D cells against cycling-induced defects, it should not be forgotten that 3D cells feature a native trap density higher than their planar counterparts (see Figure 4, left), mostly due to the GB traps not present in crystalline silicon, which may hide the initial-stage growth of cycling-induced defects. It is also interesting to note that a stronger dependence on cycling in 3D arrays was instead reported in [119], which might be ascribed to either a larger trap generation rate due to different cycling conditions or to a lower number of native traps, as fewer traps in the NAND cells would result in a more noticeable increment due to cycling. Furthermore, a transient effect related to a non-stationary condition, as hinted by the asymmetric RTN distribution there reported (see [130] for discussion) could also affect the evaluation.

**Figure 8:**
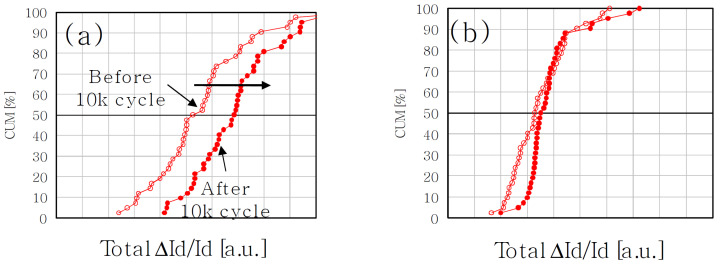
RTN cdf before and after cycling for the case of erased (**a**) and programmed (**b**) cells [110], © 2014, IEEE.

It is also interesting to note that data reported in Figure 7 were taken with programmed cells. However, in [110,131] a higher sensitivity of RTN to cycling was reported when cells are measured in the erased state (Figure 8). In the authors’ view, this result is not a consequence of a different generation rate or annealing of stress-induced traps, but rather the result of different conduction profiles of the electrons as a consequence of the charge stored in the cells, enhancing the impact of newly-created traps at the interface. Such results demonstrate that the RTN picture is still not complete, notwithstanding the excellent work put forward by the scientific community.

## 5. Conclusions

Ever since its first detection in MOS devices, RTN has retained two opposite faces, being a remarkable probe into the microscopic physics of carrier interactions with defects on one side, and a reliability threat on the other. It appears safe to say that even the transition to 3D NAND has not affected such characters, that are instead enhanced by the additional challenges built by the polycrystalline conduction channel. In this frame, this work has presented a review of the most significant experimental results in the field of random telegraph noise in 3D NAND, highlighting its current understanding and some open issues that require further efforts from the scientific and technological communities.

## Figures and Tables

**Table 1:** Range of parameter values for the acceptor-like states in the polysilicon, according to the literature (see text for references).

$N_{T}$ [cm ${}^{-3}$ eV ${}^{-1}$]	$E_{T}$ [meV]	$N_{D}$ [cm ${}^{-3}$ eV ${}^{-1}$]	$E_{D}$ [meV]
$9\times 10^{19}$–$10^{21}$	16.6–80	$1.2\times 10^{18}$–9 $\times 10^{19}$	80–500
